# Report of the Buffalo Asylum for Widows, Lying-in-Women and Foundlings

**Published:** 1856-02

**Authors:** Rosaline Brown


The following will possess interest with most of our readers:

*Report of the Buffalo Asylum for Widows, Lying-in-women and Foundlings.* — Many charitable and benevolent christians have long desired to see an institution of this kind established in "The Queen City of the Lakes." Centre of western commerce, focus of an immense travel, a thrifty and populous city, which has become "the meeting of the ways," not only for the rich and poor aspirants who pass westward to the "land of promise," but also for the sad and almost broken-hearted, who with disappointed hopes return eastward to the home of their youth, Buffalo has special need of such an institution, without which, distress and suffering not easily be described, must await the poor traveler, who is near her time, and about the penalty, "In sorrow shalt thou bring forth children."

The number of poor Foundlings, children of poverty or of crime, who have been abandoned, and found, sometimes, alas! too late, has also long proved the need of a house through which the infant's life might be safe; and poverty, and even vice, might be spared the preparation of a darker and more damning crime.

Poor widows are often, and most touchingly, marked in the holy Scriptures as objects of special charity, as objects of God's special solicitude. Those three eminent and holy charities naturally blend in one blessed work. The poor widow while she receives charity, may perhaps be able to practice charity in many slight but useful services to those who are near their time; and both may often be useful to the poor Foundling.

On the 24th of June, 1854, the Sisters of Charity began this good work; and now, at this beginning of the New Year, gratitude to their benefactors, joint founders with them of the Institution, urges them to give the following Report of their operations, its results, the benefactions received, the use of those donations, and the condition of the establishment. They have a book, a perpetual memorial, in which the names of their benefactors, and the sums, donated are inscribed; but they dare not here insert those honored names, lest they might wound the unostentatious modesty of the donors.

In a number of cottages, some five or six, so connected as to form one dwelling, with many apartments, the Sisters of Charity had to commence their labors; and even in such restricted limits, up to the first of January, 1856, they have received and taken care of 7 widows, 64 lying-in-women, and 49 foundlings. God has blessed the Institution in its care of poor women, in the painful and dangerous time of child-birth. Of 64 women, 62 were safely delivered; one passed in safety to herself through the painful time, and left the Institution in perfect health; on entering the house, she declared her belief that the child within her was already dead. The Doctor's examination confirmed the truth of her assertion. The delivery of a still-born child, already far gone in decay, made her safety and subsequent health more remarkable. Another, near her time, was brought into the house in the last stage of the Typhus Fever, and three days after died before her time, of that disease.

When the condition and prior suffering of many of those patients are considered, it seems wonderful that but one death should have occured among so many, and that one died before her time, of Typhus Fever which she brought into the Institution. Sixty-two children were safely delivered.

By strict economy the Sisters have been enabled to obtain such happy results, at what, particularly during the difficulties of beginning such a work, must be considered a small expenditure.

| | | |
|---|---|---|
| The support of the Institution has cost, - - - | | $2,119.92 |
| The Sisters have received by donations, - | $1,119.00 | |
| As renumeration from various sources, - - | 1,029.00 | |
| | | 2,148.00 |
| The Sisters owe in different small sums, - - - | | $228.92 |

During the year, the Sisters were forced to reject several applications, as their cabins were already too crowded. Trusting in a kind Providence and in the Christian charity of a generous public, they determined to erect a more suitable building, itself destined to be but part of an ampler whole, should means ever be furnished for perfecting the plan. A friend to the good work kindly lent them $2,000. The house, sewer, &c., will cost about $4,000. The building is now enclosed, and floored; on it and on the sewer, &c., $1,935 have been expended. Hopes are entertained of being able to use the house in the month of March.

To Dr. Jas. P. White, who has been kind and assiduous in his attendance, and most successful in his practice, the Sisters must ever be grateful; to Drs. Wm. Howell, and J. Brown, they tender their most grateful thanks, as also to the many generous persons of all denominations, who have contributed to this charity. May God bless them all. In conclusion, the Sisters will repeat the professions which they made in words, as follows, when they opened the Institution.

The Sisters of Charity have begun the good work of an Asylum for poor Widows, Lying-in-Women, and Foundlings, in temporary buildings, on the lot donated for the purpose, until a larger house can be erected.

A few of each class for whom this charity was instituted, are now inmates of the house, and enjoy its benefits. Such an Institution is eminently necessary, in so great a thoroughfare as Buffalo, but as the Sisters have no funds, no revenue, they cannot at first extend their charity as freely and as widely as they wish. They offer their kindest sisterly care to all the suffering, without distinction of religion; but they must depend for the support of the poor inmates on the generosity of those whom the God of mercy fits, by His spirit of charity, and by His blessings of prosperity, for being stewards of His divine bounty. The Sisters will gladly divide the last crust of bread with the poor, but they must obtain from the charitable even that crust in order to be able to divide it. This they promise, that none will be turned from their door, as long as they have room to receive, or means to nourish the suffering.

Sister Rosaline Brown.

